# Puff, Puff, Don’t Pass: harm reduction for cannabis use during a viral respiratory pandemic

**DOI:** 10.1186/s12954-023-00751-8

**Published:** 2023-02-25

**Authors:** Ryan D. Assaf, Marjan Javanbakht, Pamina M. Gorbach, Onyebuchi A. Arah, Steven J. Shoptaw, Ziva D. Cooper

**Affiliations:** 1 UCLA Center for Cannabis and Cannabinoids, Jane and Terry Semel Institute for Neuroscience and Human Behavior, University of California, Los Angeles, CA USA; 2 Department of Epidemiology, Fielding School of Public Health, University of California, Los Angeles (UCLA), Los Angeles, CA USA; 3 Department of Psychiatry and Biobehavioral Sciences, University of California, Los Angeles, CA USA; 4 Division of Infectious Diseases, David Geffen School of Medicine, University of California, Los Angeles, CA USA; 5 Department of Statistics, UCLA, Los Angeles, CA USA; 6 Center for Behavioral and Addiction Medicine, David Geffen School of Medicine, University of California, Los Angeles, CA USA; 7 Family Medicine and Psychiatry and Behavioral Sciences, David Geffen School of Medicine, University of California, Los Angeles, CA USA; 8 Department of Anesthesiology and Perioperative Medicine, David Geffen School of Medicine, University of California, Los Angeles, CA USA

**Keywords:** Cannabis, Sharing, Prepared, Paraphernalia, Inhaled, COVID-19, Substance use, Viral respiratory infection, Pandemic

## Abstract

**Background:**

Prior to the COVID-19 pandemic, cannabis use social practices often involved sharing prepared cannabis (joints/blunts/cigarettes) and cannabis-related paraphernalia. Previous studies have demonstrated that sharing paraphernalia for cannabis, tobacco, and crack cocaine is a risk factor for respiratory viral and bacterial infections. Although COVID-19 is a respiratory viral infection that spreads through droplets and airborne transmission, it is unclear if many individuals adopted harm reduction practices around sharing cannabis. This study: quantifies the prevalence of sharing prepared non-medical cannabis and cannabis-related paraphernalia reported before and during the pandemic; assesses changes in sharing of non-medical cannabis from before to during the pandemic; assess the association between frequency of non-medical cannabis use and sharing of cannabis during the pandemic; and describes how respondents obtained their cannabis and the reasons for changing their cannabis use during the pandemic to explain differences in sharing patterns.

**Methods:**

This cross-sectional study used data collected from an anonymous, US-based web survey on cannabis-related behaviors from August to September 2020 (*n* = 1833). Participants were included if they reported using a mode of inhalation for non-medical cannabis consumption. We calculated proportional changes in sharing cannabis before/during the COVID-19 pandemic. Associations between frequency of cannabis use and cannabis sharing during the COVID-19 pandemic were assessed using logistic regression analysis.

**Results:**

Overall, 1,112 participants reported non-medical cannabis use; 925 (83.2%) reported a mode of cannabis inhalation. More respondents reported no sharing during (24.9%) than before the pandemic (12.4%; *p* < 0.01); less respondents shared most of the time (19.5% before; 11.2% during; *p* < 0.01) and always during the pandemic (5.2% before; 3.1% during; *p* < 0.01)*.* After adjusting for covariates, the odds of any sharing during the pandemic for those who reported ≥ weekly cannabis use was 0.53 (95% CI 0.38, 0.75) compared to those who reported ≤ monthly.

**Conclusions:**

Sharing of prepared cannabis and cannabis-related paraphernalia decreased during the COVID-19 pandemic compared to before the pandemic. This finding suggests potential risk mitigation strategies taken by participants for COVID-19 prevention either directly through behavior change or indirectly through adherence to COVID-19 prevention recommendations. Harm reduction messaging around sharing of cannabis during surges of COVID-19 or other respiratory infections may provide benefit in reducing infection among those who use cannabis, especially as cannabis use in the USA continues to increase.

**Supplementary Information:**

The online version contains supplementary material available at 10.1186/s12954-023-00751-8.

## Introduction

Cannabis use social practices before the coronavirus disease 2019 (COVID-19) pandemic involved using and/or sharing inhaled cannabis (having more than one person put the same device or products in their mouth to inhale) with friends and sometimes with strangers [1–5]. The COVID-19 pandemic emerged at the end of December 2019, with the first cases in the USA detected from January 21 to February 23, 2020. By mid-March 2020, all 50 states had reported cases of COVID-19 [6–8]. SARS-CoV-2, the virus that causes COVID-19, leads to upper and lower respiratory infections, and is spread primarily through droplets and airborne transmission [6–10]. Given this mode of transmission, newspaper articles and social media community forums adopted the slogan “Puff, Puff, Don’t Pass” early during the pandemic (March–September 2020) to discourage sharing of prepared cannabis (joints/blunts) and cannabis-related paraphernalia with others while proposing alternative ways of going about cannabis inhalation in social settings [i.e.,^1^, ^11^]. “Puff, Puff, Don’t Pass” references smoking/inhaling cannabis, but not “passing” or sharing the same paraphernalia or product with others.

Cannabis can be inhaled with prepared cigarettes (joint/blunts), or with pipes, water pipes (bongs), cannabis vaporizers, e-cigarettes (vapes) for cannabis extracts, and rigs for highly concentrated cannabis extracts (wax/dabs). Other modes of cannabis use include oral (edibles, capsules, tinctures) and topical (balms, lotions, patches, sprays) use. However, inhaled cannabis is the most popular mode of use in the USA [12, 13]. Before the pandemic, about 17.5% of the US population (age > 12) reported past year cannabis use in 2019 [14]. There have been conflicting results on the frequency of cannabis use during the pandemic [15, 16]. One study in Spain found that individuals reported decreases in the average joints (a rolled cannabis cigarette) smoked per week during the pandemic compared to before [17]. On the other hand, two scoping reviews on cannabis use during the pandemic and other studies in the USA reported comparable frequencies of cannabis use before and during the pandemic among adults who used cannabis in the last year [15, 16, 18, 19]. It was also reported that mode of cannabis use stayed the same between the time periods with most respondents reporting a method for cannabis inhalation (smoking [joint/blunt/bong/pipe], vaporizing plant, wax/dab, and/or vaping oil/concentrates) [19].

What remains unknown is how other cannabis use behaviors, such as sharing of prepared cannabis and cannabis-related paraphernalia, were impacted during the pandemic. Previous studies have demonstrated that sharing of paraphernalia for cannabis, tobacco, and crack cocaine use is a risk factor for respiratory bacterial and viral infections [20–30]. For example, one study showed that a cluster of tuberculosis cases had been linked to sharing of a cannabis water pipe [20]. In the context of COVID-19, sharing prepared cannabis and cannabis-related paraphernalia may increase risk through direct contact with oral fluids. Decreases in sharing during the pandemic may be a harm reduction strategy for COVID-19 [27–30]. Thus, one plausible hypothesis for reductions in joint smoking among respondents in Spain may have been due to changes in social settings and sharing of joints.

In this paper, we define sharing of cannabis as sharing (having more than one person put the same device or products in their mouth to inhale) of prepared cannabis (cigarettes/joints/blunts) and cannabis-related paraphernalia (pipes/water pipes/rigs/vaporizers/ e-cigarettes/vapes) used for inhalation. With cannabis slogans such as “Puff, Puff, Don’t Pass” in March–May 2020, it is important to assess how individuals in the USA shared prepared cannabis and cannabis-related paraphernalia during the pandemic and if any risk mitigation to COVID-19 occurred [1, 11]. This study aims to: (1) quantify the prevalence of sharing prepared non-medical cannabis and cannabis-related paraphernalia before and during the pandemic; (2) assess changes in sharing of non-medical cannabis from before to during the pandemic; (3) assess the association between frequency of non-medical cannabis use and sharing of cannabis during the pandemic; and (4) describe how respondents obtained their cannabis and the reasons for changing their cannabis use during the pandemic to explain differences in sharing patterns. We hypothesize that sharing of cannabis will decrease during the pandemic compared to before and that frequency of cannabis use will be associated with sharing of cannabis. This study focuses on non-medical cannabis as this may be used recreationally or with others in comparison with cannabis for medical reasons.

## Methods

### Study design

This cross-sectional study used data from an anonymous, USA-based web survey on cannabis-related behaviors from August to September 2020. Detailed methods of the survey have been described elsewhere [19]. Briefly, respondents were eligible to complete the survey if they were (1) 18 years of age or older, (2) reported any cannabis or cannabidiol (CBD) use in the last 12 months and (3) lived in the USA (*N* = 1883). In this specific study, participants were included if they reported non-medical cannabis use and self-reported using cannabis in the following ways: smoking (joint/blunt/bong/pipe); vaporizing plant; wax/dab, and/or vaping oil/concentrates. Recruitment was based on a convenience sample of users on internet-based platforms including Reddit, Bluelight (forum for illicit drug use), Craigslist, and Twitter. The study advertisement stated the following: “Have you used cannabis (marijuana) or CBD (cannabidiol) in the past year? Participate in a UCLA survey ($5 gift card)”. Duplicate responses or “ballot stuffing” was restricted by limiting one response for each unique internet protocol (IP) address. The survey took approximately 20 to 30 min to complete, and participants were remunerated $5 for completing the survey.

### Data collection

Survey questions on non-medical cannabis use behaviors included recall for two 3-month time periods based on the World Health Organization Alcohol, Smoking, and Substance Involvement Screening Test (WHO-ASSIST): before the COVID-19 pandemic (January to mid-March 2020) and during the COVID-19 pandemic (past 3 months at the time of survey ~ June to August 2020) [31]. Questions for each time period were asked separately in this single survey. Cannabis use behavior questions included frequency of use, mode of use, sharing of prepared cannabis and cannabis-related paraphernalia, and reasons for use. We also collected demographic information including age, sex, race/ethnicity, education, sexual orientation, and state of residence.

### Ethics

This study received institutional review board approval from the University of California, Los Angeles (IRB#20-001164). All respondents received online informed consent.

### Variable definitions

The primary outcomes were sharing of prepared cannabis and cannabis-related paraphernalia before the COVID-19 pandemic and during the pandemic and the change in cannabis sharing between these two periods. Respondents used a Likert scale to agree with the following statement “I shared joints, blunts, bongs, pipes, vaporizers, or vape-pens used for cannabis (marijuana)”, with answer choices being *never, sometimes, about half the time, most of the time, or always*.

Respondents were asked to answer this question for the periods before the pandemic and during the pandemic. We coded change in sharing as an increase if there was an “increase” in sharing between the two time periods, “stayed the same” if sharing remained the same, and “decreased” if sharing decreased between time points. Finally, we dichotomized sharing of cannabis during the pandemic as “any sharing” and “no sharing”.

Additional variables included age, sex, education, sexual orientation, US Census region, state’s cannabis regulation status, and frequency of non-medical cannabis use before and during the COVID-19 pandemic. Age was kept as a continuous variable in our analysis. To capture meaningful increases in age, we rescaled the variable from 1-year increases to 10-year increases. Sex was asked as "What is your sex?” with response options: male, female, other, decline to answer. Education was categorized as high school or less, some college but no degree, and associates’ degree or higher. Sexual orientation was dichotomized with ‘sexual minority’, indicting if respondents self-identified as lesbian, gay, bisexual, or other and ‘non-sexual minority’ otherwise. The state’s cannabis regulation status was categorized as regulated (adult-use), medical only, and unregulated (CBD or fully illegal) [32]. Frequency of non-medical cannabis use was assessed for the 3-month period before (January 2020 to mid-March 2020) the pandemic and 3-month period during the pandemic. Participants were asked, “…I used non-medical cannabis (marijuana):” with answer choices never, once or twice, monthly, weekly, and daily or almost daily. Frequency of non-medical cannabis use before and during the COVID-19 pandemic was then dichotomized as once or twice/monthly (≤ monthly) use and weekly/daily (≥ weekly) use.

### Statistical analyses

We calculated frequency distributions and mean/standard deviation for demographics and cannabis use behaviors by non-medical cannabis use and those who reported a mode of inhalation for cannabis use (as smoking [joint/blunt/bong/pipe], vaporizing plant, wax/dab, and/or vaping oil/concentrates).

Furthermore, we assessed proportional changes in sharing of prepared cannabis and cannabis-related paraphernalia before and during the COVID-19 pandemic overall, by cannabis frequency of use, and by sex. Changes in sharing were evaluated using McNemar’s test for paired data by examining discordance between reported sharing before and during the pandemic for each level of sharing (i.e., discordance in no sharing before and during) with an alpha of 0.05. We were unable to compute a *p* value using McNemar’s test for paired data when the sample size for at least one discordant pair was less than 5. Finally, we examined associations between the frequency of cannabis use on cannabis sharing during the COVID-19 pandemic using logistic regression analysis. Logistic regression analyses were used to compute odds ratios, each of which was the ratio of the odds of an outcome (i.e., the probability of the outcome divided by the complement of the probability of that outcome) in the presence of a covariate to the odds of that outcome in the absence of that covariate. In our study, the outcome of interest is sharing of cannabis during the pandemic among those who use cannabis more frequently (≥ weekly) compared to those who use less frequent (≤ monthly).

Finally, we describe how respondents obtained their cannabis and the reasons for changing their cannabis use during the COVID-19 pandemic to partially explain differences in sharing patterns. To do so, we calculated frequency distributions and conducted Chi-squared analyses to compare where respondents obtained their cannabis and reasons for change by frequency of cannabis use during the pandemic and by cannabis sharing during the pandemic.

Missing data on our outcome, sharing of cannabis, was minimal in our dataset. Sixteen respondents (1.6%) had missing information on sharing of cannabis before the pandemic, and 27 respondents (2.7%) had missing information on sharing of cannabis during the pandemic. Thus, we conducted complete case analyses. All analyses were performed using SAS software version 9.4 (SAS Institute Inc., Cary, NC, USA).

## Results

In our study, 1,112 respondents reported non-medical cannabis use. Of which, 925 (83.2%) reported a mode of inhalation for non-medical cannabis use as smoking (joint/blunt/bong/pipe), vaporizing plant, wax/dab, and/or vaping oil/concentrates before and during the COVID-19 pandemic and were included in the study. Specifically, 990 individuals reported a mode of inhalation before the pandemic and 975 reported a mode of inhalation during the pandemic. The mean age of those who reported a mode of inhalation for cannabis use was 33.4 years (standard deviation 9.3). These respondents were mostly male (66.6%), non-Hispanic White (56.0%), and relatively well educated with more than half reporting an associate’s degree or higher (57.3%). Moreover, respondents were mostly from the West (38.4%), the South (30.1%), and from states that had fully regulated cannabis (49.7%) (Table 1).

**Table 1:** Demographics among those reporting non-medical cannabis and a mode of inhalation for cannabis use

	Non-medical cannabis use	Reported a mode of inhalation for cannabis*
	*n* (%)**	*n* (%)**
Total	1112 (100.0)	925 (100.0)
*Demographics*		
Age, years		
Mean (standard deviation)	33.41 (9.35)	33.35 (9.30)
Sex		
Female	374 (34.09)	306 (33.37)
Male	723 (65.91)	611 (66.63)
Sexual orientation***		
Sexual minority	340 (31.14)	275 (30.12)
Non-sexual minority	752 (68.86)	638 (69.88)
Race/ethnicity		
Hispanic/Latino	330 (30.47)	272 (30.02)
Non-Hispanic Asian	24 (2.22)	17 (1.88)
Non-Hispanic Black	94 (8.68)	77 (8.50)
Non-Hispanic American Indian or Alaska Native	18 (1.66)	12 (1.32)
Non-Hispanic Native Hawaiian or Pacific Islander	7 (0.65)	5 (0.55)
Non-Hispanic White	592 (54.66)	507 (55.96)
Non-Hispanic Other****	18 (1.66)	16 (1.77)
Education		
Less than High School	107 (9.76)	85 (9.28)
High school	144 (13.14)	123 (13.43)
Some college credit, no degree	218 (19.89)	183 (19.98)
Associates degree	272 (24.82)	229 (25.00)
College graduate or higher	355 (32.39)	296 (32.31)
US Census Region		
West	417 (37.50)	355 (38.38)
Midwest	137 (12.32)	111 (12.00)
Northeast	215 (19.33)	181 (19.57)
South	343 (30.85)	278 (30.05)
State's Cannabis Regulation Status		
Fully regulated	535 (48.11)	460 (49.73)
Medical only	326 (29.32)	259 (28.00)
CBD only	238 (21.40)	197 (21.30)
Unregulated	13 (1.17)	9 (0.97)

^*^Mode of inhalation based on check all that apply for smoking (joint/blunt/bong/pipe), vaporizing plant, vaping oil/concentrates, or wax/dab

^**^May not add to 100% because of missing data

^***^Sexual minority = Gay, lesbian, bisexual, queer, other; non-sexual minority = Straight/heterosexual

^****^Non-Hispanic Other = those who reported other race or two or more races

### Changes in sharing

**Fig. 1:**
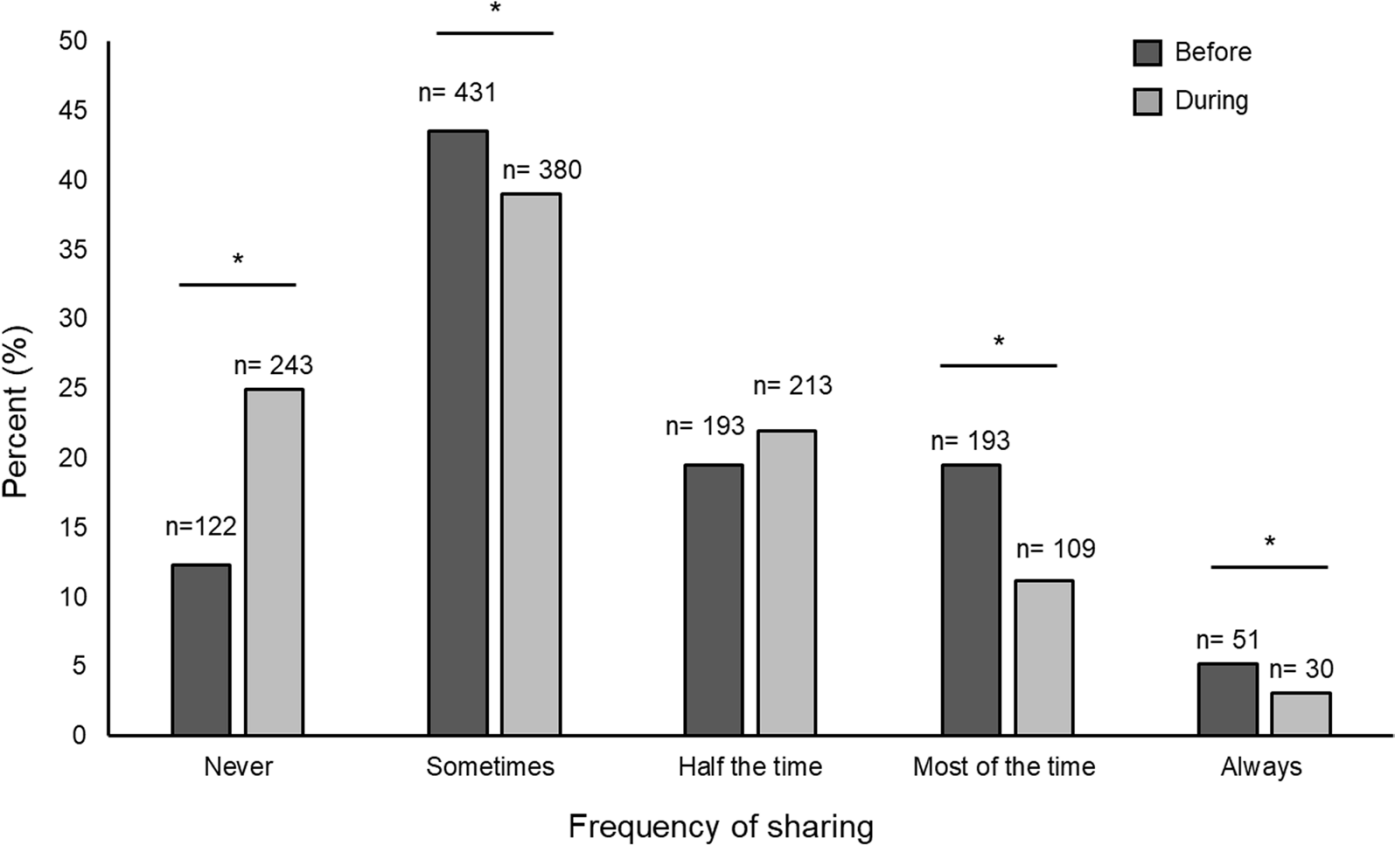
Sharing of prepared cannabis and cannabis-related paraphernalia before and during the COVID-19 pandemic. Data were collected on those who reported cannabis in the United States in a single web-based survey to assess cannabis behaviors before and during the COVID-19 pandemic. Data are presented as percent frequency distributions for sharing of prepared non-medical cannabis and cannabis-related paraphernalia before and during the pandemic. Changes in sharing were evaluated using McNemar’s test for paired data by examining discordance between reported sharing before and during the pandemic for each level of sharing (i.e., discordance in no sharing before and during). *P* values < 0.05 are indicated with an asterisk (*). Sample size for sharing cannabis was 925

Overall, there was shift in sharing during the pandemic compared to before. More respondents reported no sharing during the pandemic (12.4% before the pandemic compared to 24.9% during; *p* < 0.01). Likewise, less respondents reported sharing most of the time (19.5% before compared to 11.2% during; *p* < 0.01) and always (5.2% before compared to 3.1% during; *p* < 0.01) (Fig. 1; Additional file 1: Table S1). Detailed shifts for each level are shown in Additional file 1: Table S2.

**Fig. 2:**
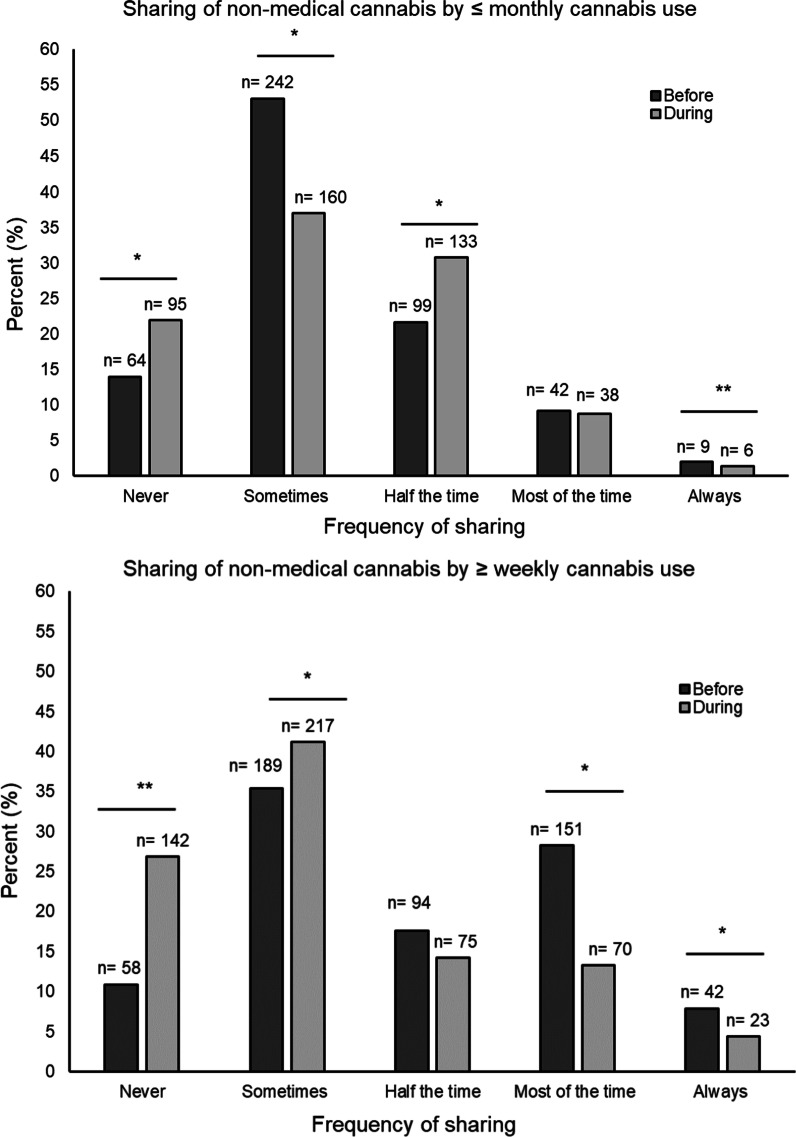
Sharing cannabis before and during the COVID-19 pandemic across levels of cannabis use. Data presented as the percent frequency distributions for sharing of prepared non-medical cannabis and cannabis-related paraphernalia before and during the pandemic across levels of non-medical cannabis frequency of use before the pandemic. Frequency of non-medical cannabis use was dichotomized as once or twice/monthly (≤ monthly) use and weekly/daily (≥ weekly) use. Changes in sharing were evaluated using McNemar’s test for paired data by examining discordance between reported sharing before and during the pandemic for each level of sharing (i.e., discordance in no sharing before and during). *P* values < 0.05 are indicated with an asterisk (*). A *p* value using McNemar’s test for paired data for some categories was not computed when the sample size for at least one discordant pair was less than 5 is indicated by a double asterisk (**). Sample size of sharing cannabis among those reporting ≤ monthly use was 419 and 506 for those reporting ≥ weekly/daily

**Fig. 3:**
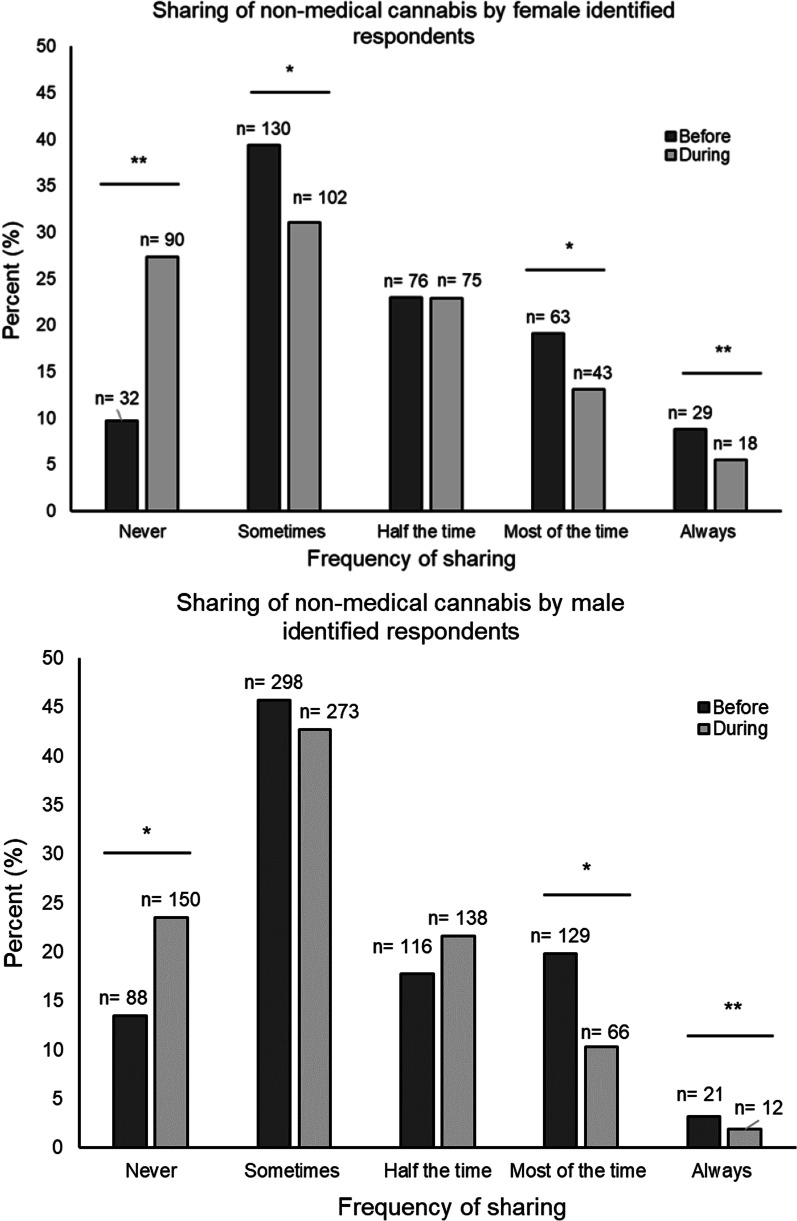
Sharing of cannabis before and during the COVID-19 pandemic across levels of sex. Data presented as percent frequency distributions for sharing of prepared non-medical cannabis and cannabis-related paraphernalia before and during the pandemic across levels of self-reported sex. Changes in sharing were evaluated using McNemar’s test for paired data by examining discordance between reported sharing before and during the pandemic for each level of sharing (i.e., discordance in no sharing before and during). *P* values < 0.05 are indicated with an asterisk (*). A *p* value using McNemar’s test for paired data for some categories was not computed when the sample size for at least one discordant pair was less than 5 is indicated by a double asterisk (**). Sample size of sharing cannabis among females was 306 and 611 for males

There were similar patterns in changes of cannabis sharing during the pandemic within subgroups of non-medical cannabis frequency of use. Among those who reported using cannabis ≤ monthly, 22% reported no sharing during the pandemic compared to 14.0% before the pandemic (*p* < 0.01); 8.8% reported sharing most of the time and 1.4% reported always sharing during the pandemic compared to 9.2% and 2.0% before the pandemic, respectively (*p value most of the time* = *0.7; discordant pair sample less than 5 and could not compute p value for always)*. Among those who reported using cannabis ≥ weekly, 26.9% reported no sharing during the pandemic compared to 10.9% before the pandemic (*p* < 0.01); 13.3% reported sharing most of the time and 4.4% reported always sharing during the pandemic compared to 28.3% and 7.9% before the pandemic, respectively (*p value most of the time* < *0.01; p value always* < *0.01)* (Fig. 2). Likewise, these patterns in changes of cannabis sharing during the pandemic were noted by sex. Among female respondents, 27.4% reported no sharing during the pandemic compared to 9.7% before the pandemic (*discordant pair sample less than 5 and could not compute p value)*. Among male respondents, 23.5% reported no sharing during the pandemic compared to 13.5% before the pandemic (*p* < 0.01) (Fig. 3).

### Frequency of cannabis use on sharing

Frequency of non-medical cannabis use (≥ weekly compared to ≤ monthly) during the pandemic was associated with sharing during the pandemic. In an unadjusted model, the odds of any sharing during the pandemic for those who reported ≥ weekly cannabis use were 0.57 (95% confidence interval [CI] 0.42, 0.78) compared to those who reported ≤ monthly during the pandemic. After adjusting for age, sex, sexual orientation, education, and state’s cannabis regulation status, the odds of any sharing during the pandemic for those who reported ≥ weekly cannabis use were 0.53 (95% CI 0.38, 0.75) compared to those who reported ≤ monthly during the pandemic. Moreover, 10-year increases in age decreased the odds of any sharing of cannabis during the pandemic in the unadjusted and adjusted models. On the other hand, self-identifying as a sexual minority and living in states with Medical only or CBD only/unregulated cannabis policies increased the odds of sharing during the pandemic in both the unadjusted and adjusted models. Finally, living in the Midwest, Northeast, and South increased the odds of sharing of prepared cannabis and cannabis-related paraphernalia during the pandemic (Table 2).

**Table 2:** Unadjusted and adjusted logistic regression for sharing of prepared cannabis during the COVID-19 pandemic (*n* = 975)

	Any reported sharing during COVID-19*	Any reported sharing during COVID-19	Any reported sharing during COVID-19 ***
	*n* (%)	Unadjusted (95% CI)^a^	Adjusted (95% CI)^b^
Cannabis frequency of use during COVID-19			
≥ Weekly	417 (71.04)	0.57 (0.42, 0.78)	0.53 (0.38, 0.75)
≤ Monthly (ref)	315 (81.19)	–	–
Age**			
Mean (SD)	32.52 (7.89)	0.72 (0.63, 0.84)	0.78 (0.66, 0.89)
Min/max	18/77	–	–
Sample	726 (74.92)	–	–
Sex			
Female	238 (72.56)	0.81 (0.60, 1.10)	0.83 (0.61, 1.14)
Male (ref)	489 (76.53)	–	–
Sexual orientation			
Sexual minority	237 (80.61)	1.56 (1.11, 2.18)	1.58 (1.19, 2.25)
Non-sexual minority	486 (72.75)	–	–
Education			
High School or Less	156 (70.91)	0.66 (0.47, 0.95)	0.55 (0.37, 0.80)
Some College	137 (70.62)	0.65 (0.45, 0.95)	0.59 (0.40, 0.87)
Associates or higher (ref)	434 (78.62)	–	–
State's cannabis regulation status			
Medical only	216 (78.55)	1.61 (1.14, 2.28)	1.56 (1.09, 2.25)
CBD Only/unregulated	184 (82.88)	2.13 (1.43, 3.18)	1.97 (1.30, 3.00)
Fully regulated (ref)	332 (69.46)	–	–
United States Census region			
Midwest	90 (76.27)	1.67 (1.04, 2.69)	–
Northeast	147 (77.78)	1.82 (1.22, 2.73)	–
South	251 (84.51)	2.84 (1.94, 4.16)	–
West (ref)	244 (65.77)	–	–

*CI* confidence interval, *SD* standard deviation, *CBD* cannabidiol,  ≥ Weekly = weekly/daily; ≤ Monthly = once or twice/monthly

^*^Row percent for any sharing of prepared cannabis and cannabis-related paraphernalia

^**^Age recentered at mean age (33 years) and rescaled per 10-unit increase

^a^Unadjusted logistic regression modeling odds for any sharing of prepared cannabis and cannabis-related paraphernalia to odds of no sharing during the COVID-19 pandemic

^b^Modeling frequency of cannabis use on sharing of prepared cannabis and cannabis-related paraphernalia during the COVID-19 pandemic adjusting for age, sex, sexual orientation, education, and state's cannabis regulation status

### How cannabis obtained and reasons for change

Last, we assessed differences in where non-medical cannabis was obtained during the pandemic and reasons for changes in cannabis use during the pandemic to better describe differences in sharing of cannabis and frequency of cannabis use. A larger proportion of those who used cannabis ≥ weekly obtained their cannabis from a regulated dispensary most of the time (38.7%) compared to 32.5% of those who used ≤ monthly (*p* < 0.01). Differences were seen for unregulated dispensaries, delivery and online services, and friends or family between those who used cannabis ≥ weekly and those who used ≤ monthly (*p* < 0.05). Additionally, a larger proportion of those who used cannabis ≥ weekly increased their use because of social distancing measures (61.9%) and time at home (81.3%) compared to those reporting ≤ monthly use (38.0% and 46.1%, respectively; *p* < 0.01).

A larger proportion of those who did not share cannabis during the pandemic obtained their cannabis from regulated dispensaries (40.0%) most of the time compared to 36.3% of those who reported any sharing (*p* < 0.01). On the other hand, a larger proportion of those who shared cannabis obtained their cannabis from friends or family most of the time (36.0%) compared to 27.4% of those who did not share cannabis during the pandemic (*p* < 0.01). Finally, a larger proportion of those who did no share reported increasing their cannabis use because of time at home (79.0%) compared to 68.7% of those reporting any sharing (*p* = 0.06) (Table 3).

**Table 3:** Ways of obtaining non-medical cannabis and reasons for changes in use during the COVID-19 pandemic

	Cannabis use during pandemic			Sharing of cannabis during pandemic		
	≥ Weekly	≤ Monthly	*p* value	No sharing	Any sharing	*p* value
	*n* (%)*	*n* (%)*		*n* (%)*	*n* (%)*	
*Where respondents obtained non-medical cannabis during the pandemic*						
Regulated dispensary			< 0.01			< 0.01
Most of the time	194 (38.65)	112 (32.46)		72 (40.00)	215 (36.26)	
Sometimes	134 (26.69)	135 (39.13)		26 (14.44)	212 (35.75)	
Never	174 (34.66)	98 (28.41)		82 (45.56)	166 (27.99)	
Unregulated dispensary						
Most of the time	125 (28.94)	134 (37.22)	< 0.01	16 (11.59)	223 (37.93)	< 0.01
Sometimes	160 (37.04)	176 (48.89)		64 (46.38)	242 (41.16)	
Never	147 (34.03)	50 (13.89)		58 (42.03)	123 (20.92)	
Delivery			< 0.01			0.8
Most of the time	115 (25.33)	110 (31.25)		38 (26.03)	163 (27.91)	
Sometimes	218 (48.02)	200 (56.82)		75 (51.37)	303 (51.88)	
Never	121 (26.65)	42 (11.93)		33 (22.60)	118 (20.21)	
Online			< 0.01			< 0.01
Most of the time	134 (30.95)	83 (24.13)		40 (27.21)	162 (28.77)	
Sometimes	193 (44.57)	200 (58.14)		55 (37.41)	300 (53.29)	
Never	106 (24.48)	61 (17.73)		51 (35.37)	101 (17.94)	
Friends/family			0.03			< 0.01
Most of the time	184 (36.95)	115 (31.34)		46 (27.38)	223 (35.97)	
Sometimes	209 (41.97)	148 (40.33)		57 (33.93)	274 (44.19)	
Never	105 (21.08)	104 (28.34)		65 (38.69)	123 (19.84)	
*Reasons for changes in cannabis use during the pandemic*						
Availability			< 0.01			0.05
Increase	128 (61.54)	90 (45.00)		30 (44.12)	180 (57.14)	
About the same	37 (17.79)	67 (33.50)		18 (26.47)	80 (25.40)	
Decrease	43 (20.67)	43 (21.50)		20 (29.41)	55 (17.46)	
Social distancing			< 0.01			0.7
Increase	156 (61.90)	54 (38.03)		41 (56.16)	161 (54.03)	
About the same	65 (25.79)	45 (31.69)		18 (24.66)	87 (29.19)	
Decrease	31 (12.30)	43 (30.28)		14 (19.18)	50 (16.78)	
Time at home			< 0.01			0.06
Increase	278 (81.29)	76 (46.06)		109 (78.99)	226 (68.69)	
About the same	40 (11.70)	47 (28.48)		19 (13.77)	60 (18.24)	
Decrease	24 (7.02)	42 (25.45)		10 (7.25)	43 (13.07)	

≥ Weekly = weekly/daily; ≤ Monthly = once or twice/monthly

^*^May not add to 100% because of missing data

## Discussion

Our study found an overall shift in sharing of prepared cannabis and cannabis-related paraphernalia during the COVID-19 pandemic toward lower levels of sharing compared to before the pandemic. More individuals reported no cannabis sharing during the pandemic, and less reported sharing most of the time or always compared to before the pandemic. This pattern of cannabis sharing between the two time periods was also seen in subgroups of cannabis frequency of use (≥ weekly and ≤ monthly) and reported sex. Previous literature has demonstrated that sharing of paraphernalia for cannabis, tobacco, and crack cocaine use is a risk factor for respiratory bacterial and viral infections including COVID-19 [20–30]. Thus, reductions in sharing behavior during a viral respiratory pandemic may reduce one’s risk of infection. Reductions in cannabis sharing during the pandemic may point to individual risk mitigation strategies for COVID-19 infection, may have been a consequence of not seeing others, or some combination of both. To partially explain this, we assessed where individuals obtained their cannabis during the pandemic and why they may have changed their cannabis use. A smaller proportion of those reporting no sharing obtained their cannabis from friends or family compared to those who reported any sharing. Additionally, more respondents reporting no sharing indicated increased cannabis use because of time at home compared to those reporting any sharing. These differences may occur directly because of one’s risk perception toward COVID-19 or indirectly due to adherence to COVID-19 policy such as stay-at-home orders. Nonetheless, decreasing one’s behavior or opportunity to share prepared cannabis and cannabis-related paraphernalia may reduce one’s risk of infection.

Public health messaging or education on risk mitigation around sharing cannabis may offer benefits during a viral pandemic given that individuals demonstrate change of such sharing behaviors in our study. To the best of our knowledge, no risk mitigation strategies or official public health messaging toward the sharing of prepared cannabis and cannabis-related paraphernalia during the COVID-19 pandemic have been suggested in the USA. Messaging around cannabis sharing during the early stages of the pandemic (March–September 2020) primarily arose from newspaper articles and social media community threads where tag lines such as “Puff, Puff, Don’t Pass” and alternatives to sharing were proposed [i.e., 1, 11]. Education around sharing smoking paraphernalia and distribution of safe crack cocaine use kits have previously been shown to reduce sharing [21, 22]. Thus, harm reduction messaging and education toward sharing practices of prepared cannabis and cannabis-related paraphernalia like “Puff, Puff, Don’t Pass” may be helpful for risk mitigation of COVID-19 infection, especially during surges of COVID-19 [11]. There may be multiple avenues for promoting messaging for harm reduction around cannabis sharing. For instance, county public health departments may work with regulated cannabis dispensaries to provide educational material or slogans around harm reduction that could be given to the individual at the time of purchase. Messaging may also be useful with other respiratory infections, such as during the cold and influenza season.

Additionally, this study identified variables associated with sharing of cannabis during the pandemic. First, those reporting ≥ weekly cannabis use during the pandemic had lower odds of sharing than those who used cannabis ≤ monthly after adjusting for age, sex, sexual orientation, education, and state’s cannabis regulation status. This may be the case as more respondents reporting ≥ weekly cannabis use reported increasing their cannabis use because of time at home (81%) and social distancing measures (62%) compared to 46% and 38% of those reporting ≤ monthly, respectively. Differences in adherence to social distancing guidelines and stay-at-home orders may have reduced opportunities to interact with and thus share with others. Second, as age increased, the odds of any sharing during the pandemic decreased. Thus, one hypothesis is that older individuals may not share as a risk mitigation for COVID-19 given the increased risk of COVID-19 severity among older groups [33]. On the other hand, younger adults may have a lower risk perception toward COVID-19 and thus lower acceptance of COVID-19 mitigation strategies [34].

### Limitations

This study has several limitations. First, this sample was a non-random convenience sample of individuals reporting cannabis use. As such, we may not be able to generalize our results to all those who use cannabis in the USA. Second, because we do not know with whom sharing was occurring or individual’s adherence to COVID-19 prevention strategies such as staying at home, it is not possible to evaluate the respondent’s true risk of COVID-19 infection. It may be that respondents only shared with those they were otherwise close with such as family members, sexual/intimate partners, or roommates. Alternatively, sharing may have changed because of adherence to stay-at-home orders (i.e., less socialization/opportunities to share). Third, we were only able to look at one-time point that was early in the pandemic. Therefore, we cannot make conclusions on lasting changes in sharing of cannabis. Fourth, there may be individuals who evaded ballot box stuffing by changing or clearing their browser cookies, switching to a different web browser, or by using a different device. This may contribute to falsified data and multiple responses by the same individual. However, we aimed to deter this by using ballot box stuffing in Qualtrics. Finally, there may be considerations such as socioeconomic, political, and environmental factors, or ever-changing public health messaging around COVID-19 within the geographic regions studied that may have led to various sharing of cannabis during the pandemic that is not measured.

## Conclusion

There was an overall decrease in sharing of prepared cannabis and cannabis-related paraphernalia during the COVID-19 pandemic compared to before the pandemic. This finding suggests potential risk mitigation strategies taken by participants for COVID-19 prevention either directly through behavior change, indirectly through adherence to COVID-19 prevention recommendations, or a combination of both. Thus, harm reduction messaging around sharing of prepared cannabis and cannabis-related paraphernalia during surges of COVID-19 and other respiratory infections may provide benefit in reducing infection incidence among those who use cannabis, especially as cannabis use continues to rise in the USA. Future studies should evaluate risk reduction in COVID-19 infection or other respiratory infections from sharing of prepared cannabis and cannabis-related paraphernalia. Additional studies should also investigate factors that relate to sharing of cannabis.

## Supplementary Information

**Additional file 1: Table S1.** Discordance in sharing of prepared cannabis and cannabis-related paraphernalia before and during the COVID-19 pandemic. **Table S2.** Changes in sharing of prepared cannabis and cannabis-related paraphernalia before and during the COVID-19 pandemic by number of transitions in change (N=925).

## Data Availability

The datasets used and/or analyzed during the current study are available from the corresponding author on reasonable request.

## Abbreviations

COVID-19: Coronavirus disease 2019
USA: United States
SD: Standard deviation
Sharing of cannabis: Sharing (having more than one person put the same device or products in their mouth to inhale) of prepared cannabis (cigarettes/joints/blunts) and cannabis-related paraphernalia (pipes/water pipes/rigs/vaporizers/e-cigarettes/vapes)
CBD: Cannabidiol
WHO-ASSIST: World Health Organization Alcohol, Smoking, and Substance Involvement Screening Test
≤ monthly: Once or twice/monthly
≥ weekly: Weekly/daily
CI: 95% Confidence interval

## References

[1] Tschorn A. COVID-19 took the joy out of social pot-smoking. Then I found a way to bring it back. 2021. Los Angeles Times.

[2] Noack R, Hofler M, Lueken U (2011). Cannabis use patterns and their association with DSM-IV cannabis dependence and gender. Eur Addict Res.

[3] Buckner JD, Crosby RD, Silgado J, Wonderlich SA, Schmidt NB (2012). Immediate antecedents of marijuana use: an analysis from ecological momentary assessment. J Behav Ther Exp Psychiatry.

[4] Phillips KT, Phillips MM, Lalonde TL, Prince MA (2018). Does social context matter? An ecological momentary assessment study of marijuana use among college students. Addict Behav.

[5] Fleming CB, Graupensperger S, Calhoun BH, Lee CM (2022). Alcohol use motives and cannabis use among young adults: between- and within-person associations based on monthly data from a community sample. Subst Use Misuse.

[6] Harrison AG, Lin T, Wang P (2020). Mechanisms of SARS-CoV-2 transmission and pathogenesis. Trends Immunol.

[7] Team CC-R (2020). Geographic differences in COVID-19 cases, deaths, and incidence—United States, February 12-April 7, 2020. MMWR Morb Mortal Wkly Rep.

[8] Schuchat A, Team CC-R. Public health response to the initiation and spread of pandemic COVID-19 in the United States, February 24-April 21, 2020. MMWR Morb Mortal Wkly Rep. 2020;69(18):551–6. 10.15585/mmwr.mm6918e2 PMC7737947 32379733

[9] Greenhalgh T, Jimenez JL, Prather KA, Tufekci Z, Fisman D, Schooley R (2021). Ten scientific reasons in support of airborne transmission of SARS-CoV-2. Lancet.

[10] World Health Organization. World Health Organization (2021). Coronavirus disease (COVID-19): How is it transmitted? https://www.who.int/news-room/questions-and-answers/item/coronavirus-disease-covid-19-how-is-it-transmitted.

[11] Greenson T. Puff, Puff, Don't Pass. 2020. North Coast Journal of Politics, People, and Art.

[12] Spindle TR, Bonn-Miller MO, Vandrey R (2019). Changing landscape of cannabis: novel products, formulations, and methods of administration. Curr Opin Psychol.

[13] Russell C, Rueda S, Room R, Tyndall M, Fischer B (2018). Routes of administration for cannabis use—basic prevalence and related health outcomes: a scoping review and synthesis. Int J Drug Policy.

[14] Substance Abuse and Mental Health Services Administration. Key Substance Use and Mental Health Indicators in the United States: Results from the 2019 National Survey on Drug Use and Health (HHS Publication No. PEP20-07-01-001, NSDUH Series H-55). Rockville, MD: Center for Behavioral Health Statistics and Quality, Substance Abuse and Mental Health Services Administration. 2020.

[15] Chong WW, Acar ZI, West ML, Wong F (2022). A scoping review on the medical and recreational use of cannabis during the COVID-19 pandemic. Cannabis Cannabinoid Res.

[16] Bonnet U, Specka M, Roser P, Scherbaum N (2023). Cannabis use, abuse and dependence during the COVID-19 pandemic: a scoping review. J Neural Transm (Vienna).

[17] Salles J, Yrondi A, Marhar F, Andant N, Dorlhiac RA, Quach B (2021). Changes in cannabis consumption during the global COVID-19 lockdown: the International COVISTRESS Study. Front Psychiatry.

[18] Brenneke SG, Nordeck CD, Riehm KE, Schmid I, Tormohlen KN, Smail EJ (2022). Trends in cannabis use among U.S. adults amid the COVID-19 pandemic. Int J Drug Policy.

[19] Assaf RD, Gorbach PM, Cooper ZD (2022). Changes in medical and non-medical cannabis use among United States adults before and during the COVID-19 pandemic. Am J Drug Alcohol Abuse..

[20] Munckhof WJ, Konstantinos A, Wamsley M, Mortlock M, Gilpin C (2003). A cluster of tuberculosis associated with use of a marijuana water pipe. Int J Tuberc Lung Dis.

[21] Frankeberger J, Cepeda A, Natera-Rey G, Valdez A (2019). Safer crack kits and smoking practices: effectiveness of a harm reduction intervention among active crack users in Mexico City. Subst Use Misuse.

[22] Ivsins A, Roth E, Nakamura N, Krajden M, Fischer B (2011). Uptake, benefits of and barriers to safer crack use kit (SCUK) distribution programmes in Victoria, Canada—a qualitative exploration. Int J Drug Policy.

[23] Harris M (2020). An urgent impetus for action: safe inhalation interventions to reduce COVID-19 transmission and fatality risk among people who smoke crack cocaine in the United Kingdom. Int J Drug Policy.

[24] Balaram K, Marwaha R, Kaelber DC (2021). The effects of substance use on severe acute respiratory syndrome coronavirus infection risks and outcomes. Curr Opin Psychiatry.

[25] Knishkowy B, Amitai Y (2005). Water-pipe (narghile) smoking: an emerging health risk behavior. Pediatrics.

[26] Urkin J, Ochaion R, Peleg A (2006). Hubble bubble equals trouble: the hazards of water pipe smoking. Sci World J.

[27] Kalan ME, Taleb ZB, Fazlzadeh M, Ward KD, Maziak W. Waterpipe Tobacco smoking: a potential conduit of COVID-19. 2020. https://blogs.bmj.com/tc/2020/03/23/waterpipe-tobacco-smoking-a-potential-conduit-of-covid-19/.

[28] Barnsley K, Singh Sohal S. Covid-19 and smoking: the elephant in the room? 2020. Retrieved from https://blogs.bmj.com/tc/2020/03/24/covid-19-and-smoking-the-elephant-in-the-room/.

[29] World Health Organization. Coronavirus disease (COVID-19): Tobacco Q&A. 2020. Retrieved from https://www.who.int/news-room/questions-and-answers/item/coronavirus-disease-covid-19-tobacco.

[30] World Health Organization. Tobacco and waterpipe use increases the risk of COVID-19. 2020. Retrieved from http://www.emro.who.int/tfi/know-the-truth/tobacco-and-waterpipe-users-are-at-increased-risk-of-covid-19-infection.html.

[31] World Health Organization Department of Mental Health and Substance Dependence. WHO- Alcohol, Smoking, and Substance Involvement Screening Test (ASSIST) V3.0. Geneva, Switzerland World Health Organization Department of Mental Health and Substance Dependence. 2000.

[32] Britannica ProCon. Legal medical marijuana states and DC. https://medicalmarijuana.procon.org/legal-medical-marijuana-states-anddc/

[33] Romero Starke K, Reissig D, Petereit-Haack G, Schmauder S, Nienhaus A, Seidler A (2021). The isolated effect of age on the risk of COVID-19 severe outcomes: a systematic review with meta-analysis. BMJ Glob Health..

[34] Malik AA, McFadden SM, Elharake J, Omer SB (2020). Determinants of COVID-19 vaccine acceptance in the US. EClinicalMedicine.

