# Supplementary material for: Puff, Puff, Don’t Pass: harm reduction for cannabis use during a viral respiratory pandemic
Source: Harm Reduct J. 2023 Feb 25;20:23. doi: 10.1186/s12954-023-00751-8 (PMC9957690; doi:10.1186/s12954-023-00751-8)
Supplement: Supplementary file 1 — Additional file 1: Table S1. Discordance in sharing of prepared cannabis and cannabis-related paraphernalia before and during the COVID-19 pandemic. Table S2. Changes in sharing of prepared cannabis and cannabis-related paraphernalia before and during the COVID-19 pandemic by number of transitions in change (N=925). [file 12954_2023_751_MOESM1_ESM.docx]

| **Table Supplement 1.** Discordance in sharing of prepared cannabis and cannabis-related paraphernalia before and during the COVID-19 pandemic | | | |
| --- | --- | --- | --- |
| **Sharing of prepared cannabis and cannabis-related paraphernalia (n=925)** | | | |
|  | No sharing during | Any sharing during | P-value* |
| No sharing before | 107 (93.04) | 8 (6.96) | <0.01 |
| Any sharing before | 125 (15.43) | 685 (84.57) |  |
|  |  |  |  |
|  | Share sometimes during | Do not share sometimes during | P-value* |
| Share sometimes before | 222 (54.95) | 182 (45.05) | 0.03 |
| Do no share sometimes before | 143 (27.45) | 378 (72.55) |  |
|  |  |  |  |
|  | Share half the time during | Do not share half the time during | P-value* |
| Share half the time before | 85 (47.75) | 93 (52.25) | 0.14 |
| Do no share half the time before | 114 (15.26) | 633 (84.74) |  |
|  |  |  |  |
|  | Share most of the time during | Do not share most of the time during | P-value* |
| Share most of the time before | 56 (31.11) | 124 (68.89) | <0.01 |
| Do not share most of the time before | 49 (6.58) | 696 (93.42) |  |
|  |  |  |  |
|  | Share always during | Do not share always during | P-value* |
| Share always before | 18 (37.50) | 30 (62.50) | <0.01 |
| Do not share always before | 6 (0.68) | 871 (99.32) |  |
| *McNemar's test of discordance for sharing prepared cannabis and cannabis-related paraphernalia before and during the COVID-19 pandemic; Test performed for each level of sharing before the pandemic (i.e., discordance in no sharing before the pandemic compared to no sharing during the pandemic) | | | |

| **Table Supplement 2.** Changes in sharing of prepared cannabis and cannabis-related paraphernalia before and during the COVID-19 pandemic by number of transitions in change (N=925) | | | | | | | | | |
| --- | --- | --- | --- | --- | --- | --- | --- | --- | --- |
|  | **Change in Sharing Cannabis by Number of Transitions** | | | | | | | | |
|  | Decrease 4 levels | Decrease 3 levels | Decrease 2 levels | Decrease 1 level | Stayed the same | Increase 1 level | Increase 2 levels | Increase 3 levels | Increase 4 levels |
|  |  |  |  |  |  |  |  |  |  |
| No sharing before (n=115) | -- | -- | -- | -- | 107 (93.0%) | 4  (3.5%) | 3  (2.6%) | 0  (0.0%) | 1  (0.9%) |
|  |  |  |  |  |  |  |  |  |  |
| Share sometimes before (n=404) | -- | -- | -- | 80 (19.8%) | 222 (55.0%) | 81 (20.0%) | 20  (5.0%) | 1  (0.3%) | -- |
|  |  |  |  |  |  |  |  |  |  |
| Share half the time before (n=178) | -- | -- | 18 (10.1%) | 49 (27.5%) | 85 (47.8%) | 24 (13.5%) | 2  (1.1%) | -- | -- |
|  |  |  |  |  |  |  |  |  |  |
| Share most of the time before (n=180) | -- | 22 (12.2%) | 75 (41.7%) | 25 (13.9%) | 56 (31.1%) | 2  (1.1%) | -- | -- | -- |
|  |  |  |  |  |  |  |  |  |  |
| Share always before (n=48) | 5  (10.4%) | 15 (31.3%) | 5  (10.4%) | 5  (10.4%) | 18 (37.5%) | -- | -- | -- | -- |
